# Characterization of the physico-chemical properties of the natural habitat and in vitro culture effects on the biochemistry, proliferation and morphology of *Lemna minuta*

**DOI:** 10.1186/s12870-023-04249-0

**Published:** 2023-05-03

**Authors:** Abdellah Maissour, Mohammed Bouqadida, Hanane Oualili, Redouane El Omari, Malika Belfaiza, Kacem Makroum

**Affiliations:** Laboratory of Plant Biotechnology, Ecology and Ecosystem Valorization, URL–CNRST n°10, Faculty of Sciences, University Chouaib Doukkali, P.O. Box 20, El Jadida, M-24000 Morocco

**Keywords:** *Lemna minuta*, Synthetic growth media, Morphophysiological parameters, Biochemical parameters, In vitro cultivation

## Abstract

In this study, the ecological conditions of the natural habitat of *Lemna minuta* Kunth in Morocco were investigated, and the impact of five synthetic growth media (Murashige-Skoog (MS), Schenk-Hildebrand (SH), Hoagland medium (HM), 10X Algal Assay Procedure (AAP), and Swedish Standard Institute medium (SIS)) on the morphophysiological and biochemical parameters was analysed. The morphophysiological parameters included root length, frond surface area, and fresh weight, while the biochemical parameters included photosynthetic pigments, carbohydrates, and protein content. The study was conducted in vitro in two phases: an uncontrolled aeration system (Phase I) and a controlled aeration system (Phase II).

The results showed that the pH, conductivity, salinity, and ammonium levels in the natural habitat were within the optimal range for duckweed growth. The measured orthophosphate concentrations were higher compared to previous observations, while the recorded chemical oxygen demand values were low. The study also revealed a significant effect of the culture medium composition on the morphophysiological and biochemical parameters of the duckweed. The fresh weight biomass, relative growth rate in fronds, relative growth rate in surface area, root length, protein content, carbohydrates, chlorophyll (a), chlorophyll (b), total chlorophyll, carotenoids, and the chlorophyll (a/b) ratio were all affected by the culture medium.

The most accurate regression models described the growth index GI(F) based on time and in vitro culture conditions in both phases. In Phase I, the best models for MS, SIS, AAP, and SH media were linear, weighted quadratic, cubic, and weighted cubic, respectively. In Phase II, the best models for all growth media were linear. The time coefficients (in days) for Phase II were 0.321, 0.547, 1.232, 1.470, and 0.306 for AAP, HM, MS, SH, and SIS, respectively.

Comparing the morphophysiological and biochemical parameters of fronds from different media and analysing the regression model results showed that the SH and MS media were the best among the tested media for the in vitro culture of *L. minuta* in controlled aeration conditions. However, further research is needed to develop new synthetic media that best promote the growth and maintenance of this duckweed in long-term culture.

## Introduction

Duckweeds have gained extensive attention in recent years due to their rich content of essential and non-essential amino acids, carbohydrates, and fats [1–5]. In addition, duckweeds are a good source of secondary metabolites, including phenolic compounds (flavonoids, hydroxycinnamic acids, and tannins) and terpenoids (diterpenoid, triterpenoids, and tetraterpenoids).

Duckweeds are notable for their high content of the protein ribulose-1, 5-bisphosphate carboxylase (RuBisCO), which is a rich source of essential amino acids such as histidine, isoleucine, leucine, lysine, methionine, phenylalanine, threonine, tryptophan, and valine [5, 6]. They also contain a range of non-essential amino acids, including alanine, arginine, asparagine, aspartate, cysteine, glutamate, glutamine, glycine, proline, serine, and tyrosine [5].

The carbohydrates in duckweeds are made up of sugars, polysaccharides, and starch. The sugar content includes arabinose, fructose, fucose, galactose, glucose, mannose, raffinose, rhamnose, sucrose, and xylose [5], while the starch is composed of amylose and amylopectin, providing an additional source of energy for various organisms.

The nutrient content and metabolite composition of these tiny and amazing plants have drawn the attention of various industries, including the animal feed industry for milk cows, bulls, sheep, ducks, turkeys, rabbits and pond fish, the biofertilizer industry, the biofuel industry for bioethanol, biogas and biohydrogen [7], the biobased chemical industry for rubber, dope, and plastics [7], the cosmetic industry for cosmetic fillers and capsules [7], the pharmaceutical industry for transgenic pharmaceutical compounds such as α-2b-interferon, insulin, human growth hormone, β-glucocerebrosidase, retinoblastoma protein, p53, angiostatin, leptin, serum albumin, hemoglobin, collagen, and monoclonal antibodies [8]. Additionally, they are being explored as emerging food products for humans.

As an example of these versatile plants that have sparked interest within the duckweed community is *Lemna minuta*. This aquatic plant flourishes in environments such as ponds, lakes, and slow-moving streams. Adapted to these watery habitats, *L. minuta* displays unique morphological characteristics that enable it to thrive. The fronds of *L. minuta* are 0.8–4.0 mm in length and 0.5–2.5 mm in width, with a length-to-width ratio of 1–2. They are never pointed and typically form colonies with 1–4 fronds, although sometimes more. These plants do not exhibit a reddish color and have 1–2 layers of air spaces. A single nerve is present, without a tracheid, and is not always clearly visible. The nerve is usually not longer than the air spaces’ extension, extending no further than 70% of the distance from the node to the tip of the frond. It generally goes straight into the elongated cell tract at the base without an angle, causing only a slight asymmetry at the frond’s base. *L. minuta* occasionally produces flowers and fruits, with a style length of 0.2–0.4 mm, fruit dimensions of 0.6–1.0 mm long and 0.4–0.7 mm wide, and seeds measuring 0.40–0.55 mm long, about 0.3 mm thick, featuring 12–15 ribs [9].

However, despite these findings, there remain many knowledge gaps and challenges in the cultivation of duckweeds. In particular, the development of high-efficiency cultivation technology is crucial for the commercial implementation of duckweed-based projects. This includes the choice of culture media for optimizing plant growth and synthesized product accumulation. Addressing these research gaps is key to advancing the study of duckweed cultivation and unlocking its full potential for a range of industries.

The aim of this study is not only to characterize the ecological habitat of *L. minuta*, but also to study the impact of well-used synthetic media (namely Murashige-Skoog (MS), Schenk-Hildebrand (SH), Hoagland medium (HM), 10X-Algal Assay Procedure medium (AAP), and Swedish Standard Institute medium (SIS)) on the proliferation, morphology, and biochemical makeup of this aquatic plant in a long-term cultivation. Moreover, we investigated the impact of controlling aeration on the proliferation and the morphology of duckweeds.

## Materials and methods

### Plant material sampling and preparation

**Fig. 1:**
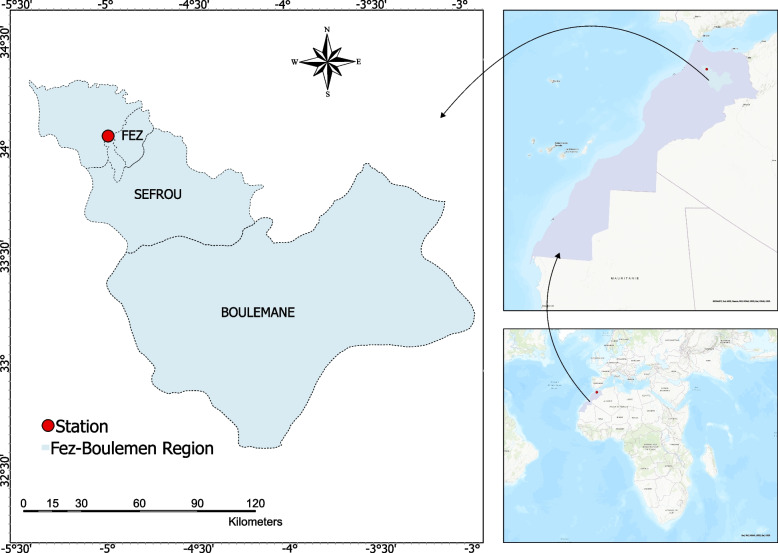
Location of the collection area *L. minuta* (prepared using ArcGIS Pro 3.0.0)

On the 20^th^ March 2022, a sample of *L. minuta* was collected from a pond at station 2 in Fez city, Morocco. This pond is situated near two other small ponds, located at stations 1 and 3, where *L. minuta* was also detected (Fig. 1). The collection of the sample was authorized by the relevant institutions and Parks and Green Spaces managers. The sample was then immediately transferred to the laboratory in airtight containers at a moderate temperature. Upon arrival at the laboratory, the sample underwent several preparatory steps before being set up for in vitro culture. These steps included rinsing the sample several times with tap water to remove any sandy elements, removing floating and attached elements such as plant debris and small aquatic animals with tweezers, and disinfecting the sample with 0.5% sodium hypochlorite solution for 30 s followed by three rinses with distilled water.

The identification of *L. minuta* was confirmed by Professor Abdellah Maissour through a comprehensive analysis that combined phytochemical screening and microscopic observations. In order to ensure the absence of anthocyanin, phytochemical screening was performed using the method described by [10]. Furthermore, *L. minuta* was identified through various microscopic observations using a trinocular light stereomicroscope (Euromex Nexius Zoom EVO, The Netherlands).

The microscopic analysis involved examining the number of fronds forming colonies, which typically range from 1 to 4, and verifying the presence of a single root per frond. Frond dimensions were measured to ensure they fell within the range of 0.8–4.0 mm long and 0.5–2.5 mm wide, with a length-to-width ratio of 1–2 times. Additionally, entire frond margins were checked, as well as the absence of reddish coloration. The presence of only one nerve, often not very distinct and rarely longer than the extension of the air spaces, was also confirmed [9]. A voucher specimen of the *collected L. minuta* has been deposited in a publicly available herbarium located in the Department of Biology, Botany and Ecology Unit, Faculty of Sciences El Jadida.

### In vitro growth culture

Fronds of *L. minuta* were aseptically placed in five synthetic growth media (250 mL) with three replicates each: Murashige-Skoog (MS) [11], Schenk-Hildebrand (SH) [12], Hoagland medium (HM) [13], 10X-Algal Assay Procedure medium (AAP) [13] and Swedish Standard Institute medium (SIS) (Table 1). During the entire 47-day experiment, duckweed cultures were maintained in a modified incubator equipped with cool fluorescent light (FOC-225E, Velp Scientifica, Milan, Italy) under a temperature of 22 °C, light intensity of 10,000 lx, and a photoperiod of 16/8 h. The growth media were renewed every week and whenever infections were detected.

**Table 1:** Composition of culture media

	**MS**	**AAP**	**SH**	**HM**	**SIS**
**Macronutrients**	KNO_3_ MgSO_4_ KH_2_PO_4_ NH_4_NO_3_ CaCl_2_	NaNO_3_ MgCl_2_.6H_2_O CaCl_2_.2H_2_0 MgSO_4_.7H_2_O KH_2_PO_4_.3H_2_O	NH_4_H_2_PO_4_ CaCl_2_.H_2_O MgSO_4_.7H_2_O KNO_3_	KNO_3_ MgSO_4_ KH_2_PO_4_ Ca(NO_3_)_2_	NaNO_3_ KH_2_PO_4_ MgSO_4_.7H_2_O CaCl_2_ Na_2_CO_3_
**Micronutrients**	H_3_BO_3_ MnSO_4_ ZnSO_4_.7H_2_O Na_2_MoO_4_.2H_2_O CuSO_4_.5H_2_O CoCl_2_ .6H_2_O	H_3_BO_3_ MnCl_2_.4H_2_O FeCl_3_.6H_2_O Na_2_EDTA.2H_2_O ZnCl_2_ CoCl_2_.6H_2_O Na_2_MoO_4_.2H_2_O CuCl_2_.2H_2_O	H_3_BO_3_ CuSO_4_.5H_2_O MnSO_4_.4H_2_O Na_2_MoO_4_.2H_2_O ZnSO_4_.7H_2_0	H_3_BO_3_ Na_2_MoO_4_.2H_2_O CuCl_2_.2H_2_O MnCl_2_.4H_2_O ZnCl_2_	H_3_BO_3_ MnCl_2_.4H_2_O Na_2_MoO_4_.2H_2_O ZnSO_4_.7H_2_O CuSO_4_.5H_2_O Co(NO_3_)_2_.6H_2_O
**Other chemical compounds**	FeSO_4_.7H_2_O Na_2_EDTA KI Nicotinic acid	NaHCO_3_	Na_2_EDTA FeSO_4_.7H_2_O Auxin Sugar	FeCl_3_.6H_2_O Na_2_EDTA	FeCl_3_.6H_2_O Na_2_EDTA.2H_2_O

**Fig. 2:**
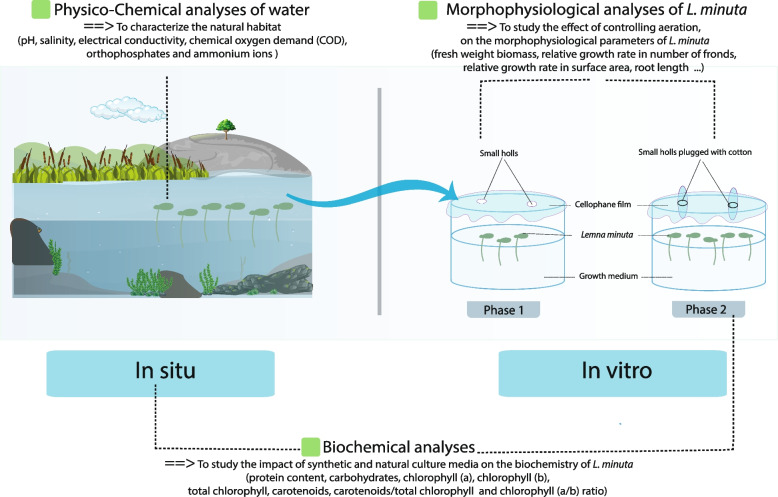
Study design

The experiment was divided into two phases (Fig. 2). Phase I (first 19 days) involved covering the growth media with perforated cellophane film, while Phase II (next 28 days) involved controlling aeration by plugging the pores in the media with absorbent cotton.

### Morphophysiological analyses (measurement of growth indices)

Morphophysiological analyses were conducted in correspondence with the two phases of growth conditions. During Phase I (first 19 days), the number and surface area of the fronds as well as the length of the roots were determined semi-automatically using Image J (Pack Fiji, v1.53 K). Phase II continued for an additional 14 days, totalling 33 days for these measurements.

After the full 47 days of the experiment, the fresh weight, another component of the morphophysiological analyses, was determined. Fronds were transferred to calibrated and perforated polystyrene tubes with small holes in their rounded bottoms. The tubes were then centrifuged at 3,000 rpm for 10 min at room temperature. The dried fronds in the tubes were reweighed, and the fresh weight was calculated by subtracting the tare weight of the tube.

The following growth indices were calculated:

- The biomass fresh weight growth index (Eq. 1)
- The frond number growth index (Eq. 2)
- The frond surface area growth index (Eq. 3)
- The relative growth rate (RGR) of frond number (Eq. 4)
- The relative growth rate (RGR) of frond surface area (Eq. 5)
  1 $$G.I_{\alpha } (P) = \frac{{p_{n} }}{{P_{0} }}$$
  2 $$G.I_{\alpha } (F) = \frac{{F_{n} }}{{F_{0} }}$$
  3 $$G.I_{\alpha } (S) = \frac{{S_{n} }}{{S_{0} }}$$
  4 $$RGR_{\alpha } (X) = \frac{{\ln (F_{N} ) - \ln (F_{0} )}}{{D_{0}^{N} }}$$
  5 $$RGR_{\alpha } (X) = \frac{{\ln (S_{N} ) - \ln (S_{0} )}}{{D_{0}^{N} }}$$

With:

$$\begin{aligned}& F_{n} : \, Final \, number \, of \, fronds \\& F_{0} : \, Initial \, number \, of \, fronds\\& P_{n} :Final \, fresh \, weight \\ & P_{0} : \, Initial \, fresh \, weight \\ & S_{n} : \, Final \, surface \\ & S_{0} : \, Initial \, surface \\ & D_{0}^{N} : \, Duration \end{aligned}$$

### Biochemical analyses

The Biochemical Analyses were conducted at the end of the experiment, specifically on the 47^th^ day, to assess protein content, carbohydrate content, and photosynthetic pigments.

The extraction of proteins from duckweed samples was performed according to the protocol described by [14], with subsequent quantification conducted using the Bradford method [15]. The quantification of chlorophylls and carotenoids was performed using the methods outlined by [16, 17]. The measurement of carbohydrates was performed using the method described by [18].

During the course of the experiment, the biochemical analysis of duckweed was conducted at two critical stages. The first analysis was performed immediately after the pre-treatment of duckweed collected from its natural habitat to assess its initial biochemical composition. The second analysis was performed after the completion of the experiment, on duckweed grown in synthetic media, to compare the impact of the synthetic media on the duckweed’s biochemical makeup.

### Characterization of the natural habitat’s physico-chemical properties

It was conducted through the measurement of pH, salinity, and electrical conductivity in situ using a Bante 900P portable multimeter (Bante Instruments; Shanghai, China). Chemical oxygen demand (COD) was determined using the potassium permanganate index, as outlined by French Standard NFT 90-101 [19]. Orthophosphates and ammonium ions present in the environment were determined and estimated, respectively, according to the French Standards NF EN ISO 6878 and NF T90-015-1 [20, 21].

### Statistical analysis

Various analytical techniques, including the generation of graphs to display growth index variations based on experimental duration, were performed using SPSS (V.26; IBM SPSS Statistics, Chicago, IL, USA). All tests were conducted as two-tailed with a significance level (α) set at 0.05. Data normality was assessed using the Shapiro-Wilk test. For normally distributed data, One-way ANOVA was applied, followed by Tukey’s post hoc test if group variances were found to be homogeneous according to the Levene test statistic. In cases where the Levene test of homogeneity of variances was significant, Welch’s ANOVA was utilized, and Dunnett’s T3 post hoc analysis was conducted to identify pairwise differences. Data with a non-normal distribution were analyzed using the Kruskal-Wallis test, followed by Dunn’s post hoc test.

To model the growth index variable as a function of cultivation time, we constructed regression equations based on the best-fitted prediction models. Before fitting the simple linear regression model, we assessed the data to ensure that it met the necessary assumptions, including linearity, independence of residuals, homoskedasticity, and the absence of outliers or highly influential cases. When these assumptions were not met, alternative regression techniques, such as polynomial regression or weighted least squares, were employed to better represent the relationship between the independent and dependent variables.

The chosen regression models were evaluated for goodness of fit and statistical significance to ensure that they provided reliable and unbiased estimates of the relationships under investigation. This comprehensive approach to statistical analysis allowed for a thorough understanding of the data and the underlying relationships within the study.

## Results

### Physico-chemical properties of the natural habitat of *L. minuta*

In the natural habitat of *L. minuta*, the pH values range between 7.2 and 7.8, conductivity ranges between 998.33 µS/cm and 1139 µS/cm (Table 2), orthophosphate concentration ranges between 0.36 and 1.26 g/L, salinity ranges between 0.48 and 0.51 psu, chemical oxygen demand (COD) values as measured by the potassium permanganate index range between 10.45 and 13.22 mg/L, and the concentration of ammonium [NH_4_^+^] ranges between 0.52 and 3.73 g/L.

**Table 2:** Physico-chemical characteristics of the natural aquatic habitat of *L. minuta*’s growth

	***Station 1***	***Station 2***	***Station 3***
***pH***	7.48 ± 0.048	7.2 ± 0.06	7.76 ± 0.03
***Conductivity (µS/cm)***	1007.33	998.33	1139
***Orthophosphates (g/L)***	0.36 ± 0.15	1.26 ± 0.15	0.66 ± 0.06
***Salinity (psu)***	0.48 ± 0.03	0.48 ± 0.01	0.51 ± 0.006
***COD (mg/L)***	10.78 ± 1.12	13.22 ± 5.83	10.45 ± 6.47
***[NH***_***4***_^**+**^***] (g/L)***	0.83 ± 0.12	3.73 ± 0.95	0.52 ± 0.19

### Morphophysiological analyses

#### Fresh weight biomass

The results showed that the samples grown in the SH medium produced the highest biomass with a median of 121.33 mg over a 47-day period, as seen in Table 3. The samples grown in the MS medium produced the second-highest biomass with a median of 110.00 mg, followed by those grown in the SIS (median = 59.00 mg) and HM (median = 54.00 mg) media. The samples grown in the AAP medium produced the lowest biomass, with a median of 47.33 mg.

**Table 3:** Variation of fresh weight biomass (in mg) of *L. minuta* in different studied culture media

	***N***		***Mean***	***Std. dev***	***Min***	***Max***	**Median**
***AAP***	3	**(a)**	47.889	1.895	46.333	50.000	47.333
***HM***	3	**(a,b)**	53.000	5.897	46.667	58.333	54.000
***MS***	3	**(a,b)**	104.889	13.418	89.667	115.000	110.000
***SH***	3	**(b)**	120.444	3.421	116.667	123.333	121.333
***SIS***	3	**(a,b)**	56.778	5.985	50.000	61.333	59.000

The different letters indicate significant differences between the groups determined by a Kruskal–Wallis analysis combined with a Dun post-test (*p* < 0.05)

A Kruskal–Wallis test revealed a significant effect of the growth medium on the production of fresh weight biomass in the studied species (H(4, *N* = 15) = 12.230, *p* = 0.016). The Dunn’s post hoc test revealed that this production is significantly greater in the SH medium compared to the AAP (*p* < 0.05), but there are no significant differences between the HM, MS, and SIS media.

#### Number of fronds

**Fig. 3:**
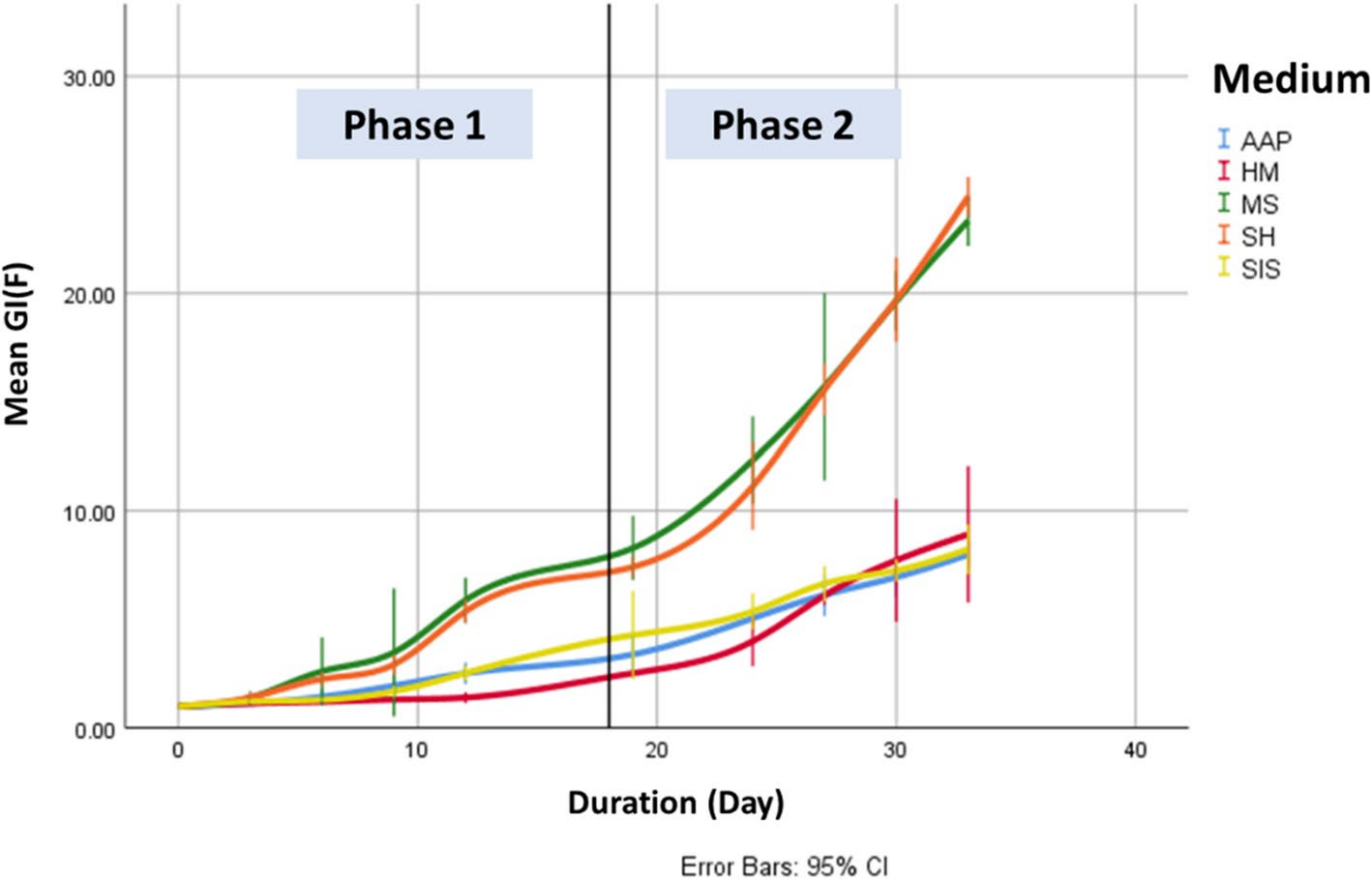
Temporal evolution of the GIα(F) index

**Fig. 4:**
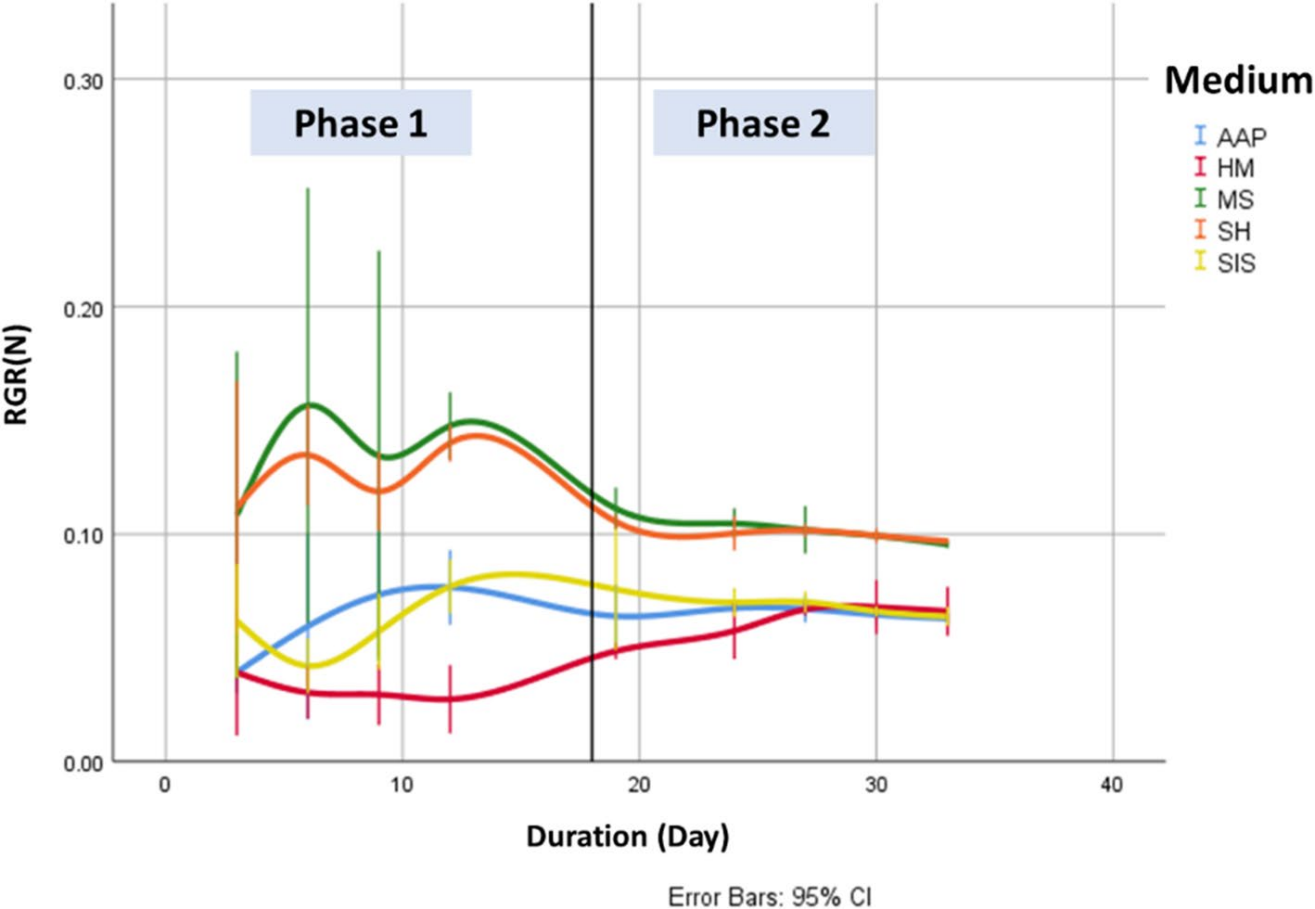
Temporal evolution of the RGRα(N) index

There was an increase in the proliferation of all fronds as the study progresses from phase I to phase II (Table 4, Figs. 3 and 4). The strongest increases in the medians of GIα(F) are seen in fronds from MS (1706%) and SH (1642%) media.

**Table 4:** Statistical characteristics of GIα(F) and RGRα(N) indices

	**N**		**Mean**	**Std. dev**	**Min**	**Max**	**Q1**	**Median**	**Q3**
**GIα(F)**									
***Phase 1***									
AAP	18	***(ab)***	1.899	0.877	1.000	3.763	1.125	1.625	2.550
HM	18	***(a)***	1.423	0.522	1.000	2.588	1.125	1.231	1.400
MS	18	***(b)***	3.773	2.697	1.000	8.975	1.400	2.806	5.950
SH	18	***(b)***	3.391	2.355	1.000	7.613	1.388	2.563	5.475
SIS	18	***(ab)***	1.993	1.199	1.000	4.763	1.225	1.456	2.563
***Phase 2***									
AAP	12	***(a)***	6.523	1.153	4.938	8.188	5.425	6.531	7.494
HM	12	***(a)***	6.681	2.073	3.475	10.188	5.156	6.644	8.281
MS	12	***(b)***	17.754	4.404	11.638	23.850	13.494	18.063	21.463
SH	12	***(b)***	17.714	5.181	10.300	24.850	13.475	17.425	22.263
SIS	12	***(a)***	6.873	1.121	5.138	8.750	6.013	6.969	7.663
**RGRα(N)**									
***Phase 1***									
AAP	15	***(a)***	0.063	0.016	0.036	0.082	0.043	0.068	0.078
HM	15	***(a)***	0.035	0.010	0.021	0.050	0.028	0.033	0.047
MS	15	***(b)***	0.132	0.030	0.078	0.200	0.109	0.126	0.149
SH	15	***(b)***	0.122	0.017	0.091	0.144	0.107	0.126	0.136
SIS	15	***(a)***	0.063	0.015	0.037	0.082	0.050	0.065	0.078
***Phase 2***									
AAP	12	***(a)***	0.065	0.002	0.062	0.069	0.063	0.066	0.067
HM	12	***(a)***	0.065	0.006	0.052	0.073	0.062	0.066	0.067
MS	12	***(b)***	0.100	0.004	0.095	0.108	0.097	0.100	0.104
SH	12	***(b)***	0.100	0.002	0.096	0.103	0.097	0.100	0.101
SIS	12	***(a)***	0.067	0.003	0.063	0.073	0.065	0.067	0.070

Significant differences among the groups are indicated by various letters, which were determined through a combination of Kruskal–Wallis analysis and Dun post-test (*p* < 0.05)

The Kruskal–Wallis test shows a significant effect of the growth media on the GIα(F) of the studied species in the two phases (Phase I: H(4, *N* = 90) = 13.870, *p* = 0.008 and Phase II: H(4, *N* = 60) = 42.679, *p* = 0.000)), and the Dunn’s post hoc test shows significant differences between groups in different growth media in all of these phases, during which fronds from MS and SH have the highest mean ranks and AAP and HM have the lowest.

The Kruskal–Wallis test shows a significant effect of the culture medium on RGRα(N) of the *L. minuta* in the two studied phases (Phase I: H(4, *N* = 75) = 60.253, *p* = 0.000 and Phase II: H(4, *N* = 60) = 43.598, *p* = 0.000) and the Dunn’s post hoc test shows significant differences between the groups of different culture media in all these phases, during which the mean ranks of the fronds of AAP and SIS are significantly different from those of MS and SH.

The best regression models obtained, describing the development of the growth index GI(F) as a function of time and in vitro culture conditions, are as follows:

In the first phase:

$$\begin{aligned} &AAP:\gamma_{{{\text{GI}}\alpha \left( {\text{F}} \right)}} = { ( - 0}{\text{.001)t}}^{3} {\text{ + (0.020)t}}^{2} { + ( - 0}{\text{.023)t + (1}}{.008) }\\ &HM:\gamma_{{{\text{GI}}\alpha \left( {\text{F}} \right)}} = {(0}{\text{.0004)t}}^{3} + {( - 0}{\text{.007)t}}^{2} { + (0}{\text{.061)t + (1}}{.000)}\\ &MS:\gamma_{{{\text{GI}}\alpha \left( {\text{F}} \right)}} = {(0}{\text{.408)t + (0}}{.438)}\\ &SH:\gamma_{{{\text{GI}}\alpha \left( {\text{F}} \right)}} = {( - 0}{\text{.002)t}}^{{3}} { + (0}{\text{.061)t}}^{{2}} { + ( - 0}{\text{.131)t + (1}}{.098)}\\ &SIS:\gamma_{{{\text{GI}}\alpha \left( {\text{F}} \right)}} = {(0}{\text{.01)t}}^{{2}} { + ( - 0}{\text{.008)t + (1}}{.018)}\\ \end{aligned}$$

- The best model was a linear regression in the MS medium with *F*(1,16) = 224.128, *p* < 0.001, *R*2 adjusted = 0.929.
- It was a weighted quadratic in the SIS medium with *F*(2,15) = 151.189, *p* < 0.001, *R*2 adjusted = 0.946.
- It was a cubic model for the AAP and SH environments with *F*(3,14) = 129.712, *p* < 0.001, and *R*2 adjusted = 0.958, and *F*(3,14) = 239.819, *p* < 0.001, and *R*2 adjusted = 0.97, respectively. However, in the HM medium, the best model was a weighted cubic model with *F*(3,14) = 615.697, *p* < 0.001, and *R*2 adjusted = 0.991.
  In the second phase:
  $$\begin{gathered} AAP:\gamma_{{{\text{GI}}\alpha \left( {\text{F}} \right)}} = {(0}{\text{.321)t + ( - 2}}{.613) } \hfill \\ HM:\gamma_{{{\text{GI}}\alpha \left( {\text{F}} \right)}} = {(0}{\text{.547)t + ( - 8}}{.899)} \hfill \\ MS:\gamma_{{{\text{GI}}\alpha \left( {\text{F}} \right)}} = {(1}{\text{.232)t + ( - 17}}{.348)} \hfill \\ SH:\gamma_{{{\text{GI}}\alpha \left( {\text{F}} \right)}} = {(1}{\text{.470)t + ( - 24}}{.185)} \hfill \\ SIS:\gamma_{{{\text{GI}}\alpha \left( {\text{F}} \right)}} = {(0}{\text{.306)t + ( - 1}}{.851)} \hfill \\ \end{gathered}$$
- The best models for all growth media were linear, with *F*(1,10) = 184.630, *p* < 0.001, *R*2 adjusted = 0.943 for AAP, *F*(1,10) = 58.299, *p* < 0.001, *R*2 adjusted = 0.839 for HM, *F*(1,10) = 240.343, *p* < 0.001, *R*2 adjusted = 0.956 for MS, *F*(1,10) = 845.327, *p* < 0.001, *R*2 adjusted = 0.987 for SH, and *F*(1,10) = 106.760, *p* < 0.001, *R*2 adjusted = 0.906 for SIS. The time coefficients (in days) were 0.321, 0.547, 1.232, 1.470, and 0.306 for the AAP, HM, MS, SH, and SIS, respectively.

#### Root length

The analysis of root length revealed that the lowest values were recorded in the fronds of MS during the first phase (Table 5). There was an increase in the root length of fronds from all media as the study progressed from phase I to phase II. The most notable increases in median values were observed in the roots of fronds from MS (5844%) and AAP (3908%) media (0.011 cm-0.627 cm in the case of MS, 0.042 cm -1.692 cm in the case of AAP).

**Table 5:** Statistical characteristics of root length (in cm) and GIα index

	**N**		**Mean**	**Std. dev**	**Min**	**Max**	**Q1**	**Median**	**Q3**
**Root length (cm)**									
***Phase 1***									
AAP	18	***(ab)***	0.046	0.018	0.026	0.076	0.031	0.042	0.059
HM	18	***(c)***	0.760	0.457	0.023	1.238	0.524	0.900	1.166
MS	18	***(a)***	0.014	0.015	0.001	0.037	0.001	0.011	0.023
SH	18	***(c)***	0.945	0.445	0.021	1.361	0.977	1.020	1.260
SIS	18	***(bc)***	0.804	0.366	0.024	1.103	0.821	0.952	1.003
***Phase 2***									
AAP	12	***(c)***	1.589	0.429	0.902	2.230	1.193	1.692	1.884
HM	12	***(ab)***	0.931	0.145	0.647	1.126	0.838	0.962	1.006
MS	12	***(a)***	0.598	0.214	0.123	0.815	0.492	0.627	0.760
SH	12	***(c)***	1.463	0.126	1.205	1.642	1.390	1.472	1.542
SIS	12	***(bc)***	1.129	0.060	1.001	1.182	1.120	1.146	1.171
***Phase 1***									
AAP	18	***(ab)***	1.689	0.652	0.968	2.961	1.169	1.513	2.113
HM	18	***(c)***	28.448	17.230	1.000	48.326	19.909	34.528	45.627
MS	18	***(a)***	0.400	0.545	0.003	1.767	0.043	0.063	1.000
SH	18	***(c)***	32.214	15.799	1.000	47.962	31.046	34.069	45.972
SIS	18	***(bc)***	26.895	12.681	1.000	41.166	26.838	30.670	33.278
**GI(Root)**									
***Phase 2***									
AAP	12	***(c)***	58.231	15.647	35.384	79.367	42.447	60.085	71.989
HM	12	***(b)***	34.554	5.843	27.350	42.970	30.077	33.129	40.413
MS	12	***(b)***	19.002	14.427	0.332	38.481	2.133	22.246	30.778
SH	12	***(ac)***	50.678	12.300	36.036	72.175	38.402	51.024	60.149
SIS	12	***(ab)***	37.990	6.599	31.698	48.253	33.605	35.134	44.788

Significant differences between the groups are indicated by distinct letters and were found through a Kruskal-Wallis analysis combined with a Dun post-test (*p* < 0.05)

During the first phase, the MS medium exhibited the shortest median root lengths, with values of 0.001 cm on days 6, 9, and 12, and slightly higher values of 0.023 cm on the 3^rd^ day and 0.029 cm on the 19^th^ day. In contrast, the AAP medium had a median root length of 0.030 cm on the 3^rd^ day, increasing to 0.076 cm by the 19^th^ day. As the study advanced to the second phase, the median root length for MS medium experienced a substantial increase, reaching 0.321 cm on the 24^th^ day, 0.611 cm on the 27^th^ day, 0.751 cm on the 30^th^ day, and 0.808 cm on the 33^rd^ day. Similarly, the AAP medium saw further growth in median root length, registering 0.981 cm on the 24^th^ day, 1.561 cm on the 27^th^ day, and peaking at 2.032 cm on the 33^rd^ day.

The Kruskal–Wallis test revealed a significant effect of culture media on the root length of this duckweed in both phases (Phase I: H(4, *N* = 90) = 52.177, *p* = 0.000 and Phase II: H(4, *N* = 60) = 46.543, *p* = 0.000), and the Dunn’s post hoc test showed significant differences between groups from different culture media in all these phases.

Throughout the study, the fronds with the lowest rank averages were those from the MS medium (12.61, and 7.50 in the first and the second phase, respectively). Conversely, the fronds with the highest rank averages were those from the SH medium (66.67) in the first phase, and those from the AAP (47) and SH (46.58) media in the second phase.

Throughout this study, the mean ranks of fronds from SH and SIS were not found to be significantly different, however, their ranks were found to be significantly different from those of fronds from MS.

The Kruskal-Wallis test revealed a significant effect of culture medium on the GIα (roots) of the duckweeds in the two phases (Phase I: H(4, *N* = 90) = 51.650, *p* = 0.000 and Phase II: H(4, *N* = 60) = 37.102, *p* = 0.000), and the Dunn’s post hoc test showed significant differences between fronds from different culture media in all phases.

#### Frond size

The Kruskal–Wallis test did not reveal any significant impact of the culture medium on frond surface area for the species studied during the first phase (H(4, *N* = 90) = 4.950, *p* = 0.292), nor the second (H(4, *N* = 60) = 5.896, *p* = 0.207) (Table 6).

**Table 6:** Statistical characteristics of surface (in cm^2^) and GIα and RGRα indices

	**N**		**Mean**	**Std. dev**	**Min**	**Max**	**Q1**	**Median**	**Q3**
**Surface (cm**^**2**^**)**									
***Phase 1***									
AAP	18	***(a)***	0.019	0.009	0.006	0.032	0.011	0.016	0.030
HM	18	***(a)***	0.027	0.015	0.001	0.055	0.021	0.025	0.035
MS	18	***(a)***	0.024	0.012	0.004	0.047	0.016	0.026	0.031
SH	18	***(a)***	0.029	0.020	0.003	0.066	0.016	0.024	0.037
SIS	18	***(a)***	0.030	0.014	0.010	0.058	0.021	0.027	0.039
***Phase 2***									
AAP	12	***(a)***	0.018	0.003	0.011	0.021	0.016	0.019	0.020
HM	12	***(a)***	0.016	0.020	0.001	0.052	0.002	0.006	0.028
MS	12	***(a)***	0.013	0.008	0.002	0.028	0.007	0.012	0.018
SH	12	***(a)***	0.032	0.031	0.007	0.082	0.008	0.012	0.069
SIS	12	***(a)***	0.017	0.010	0.006	0.033	0.008	0.013	0.026
**GIα(Surface)**									
***Phase 1***									
AAP	18	***(a)***	0.639	0.302	0.199	1.080	0.341	0.538	0.997
HM	18	***(ab)***	1.004	0.525	0.047	2.133	0.620	1.007	1.254
MS	18	***(ab)***	0.820	0.394	0.119	1.516	0.683	0.814	1.000
SH	18	***(ab)***	1.177	0.820	0.126	2.830	0.605	1.000	1.514
SIS	18	***(b)***	1.319	0.692	0.349	2.900	0.956	1.023	1.500
***Phase 2***									
AAP	12	***(a)***	0.586	0.117	0.354	0.728	0.536	0.619	0.659
HM	12	***(a)***	0.525	0.578	0.047	1.513	0.100	0.181	0.927
MS	12	***(a)***	0.417	0.220	0.079	0.780	0.259	0.348	0.641
SH	12	***(a)***	1.364	1.371	0.296	3.548	0.327	0.460	3.015
SIS	12	***(a)***	0.685	0.349	0.275	1.224	0.371	0.643	0.986
**RGRα(Surface)**									
***Phase 1***									
AAP	15	***(a)***	-0.082	0.082	-0.266	0.026	-0.123	-0.054	-0.012
HM	15	***(ab)***	-0.013	0.088	-0.161	0.174	-0.082	0.003	0.047
MS	15	***(ab)***	-0.038	0.105	-0.237	0.139	-0.095	-0.023	-0.001
SH	15	***(ab)***	-0.015	0.109	-0.230	0.148	-0.056	-0.005	0.053
SIS	15	***(b)***	0.030	0.093	-0.117	0.292	-0.015	0.022	0.056
***Phase 2***									
AAP	12	***(a)***	-0.020	0.008	-0.038	-0.010	-0.022	-0.017	-0.015
HM	12	***(a)***	-0.046	0.046	-0.128	0.015	-0.079	-0.056	-0.004
MS	12	***(a)***	-0.037	0.024	-0.094	-0.008	-0.047	-0.038	-0.017
SH	12	***(a)***	-0.008	0.038	-0.048	0.044	-0.040	-0.027	0.038
SIS	12	***(a)***	-0.018	0.021	-0.054	0.007	-0.037	-0.016	0.000

Significant differences between the groups are indicated by distinct letters and were found through a Kruskal–Wallis analysis combined with a Dun post-test (*p* < 0.05)

When transitioning from the first to the second phase, there was a decrease in the GIα (surface) index for all fronds except for those from AAP.

The Kruskal–Wallis test shows a significant effect of culture medium on the GIα (surface) index for the species studied during the first phase (H(4, *N* = 90) = 13.972, *p* = 0.007), but not during the second phase (H(4, *N* = 60) = 7.585, *p* = 0.108).

In the first phase, the Dunn’s post hoc test shows that the lowest value for GIα (surface) index is that of fronds from AAP.

The Kruskal–Wallis test shows a significant effect of culture medium on the RGRα (surface) of the studied species in the first phase (H(4, *N* = 75) = 12.070, *p* = 0.017), but not in the second phase (H(4, *N* = 60) = 7.308, *p* = 0.120). The results of this index are similar to those of the GIα (surface) index.

### Biochemical analyses

#### Protein contents

The results showed that the medium with the highest median protein content was recorded in the natural environment, with a value of 3894 µg/g (Table 7). In vitro, the samples with the highest protein content in the five synthetic media studied were those from the SIS medium, followed by those from the AAP, SH, and MS media, with medians of 40.79, 33.69, 24.92, and 23.01 µg/g fresh weight, respectively, and finally the HM medium with a mean of 21.15 µg/g fresh weight. The Kruskal–Wallis test showed a significant effect of the growth medium on the protein content of the studied species (H(5, *N* = 18) = 11.105, *p* = 0.049). However, the comparison using the Dunn’s post hoc test did not show any significant differences between the groups of different media.

**Table 7:** Variation of *L. minuta*’s protein and carbohydrate content in different studied culture media

	***N***		***Mean***	***Std.dev***	***Min***	***Max***	***Median***
**Proteins (µg/g FW)**							
AAP	3	**(a)**	39.21	9.77	33.44	50.49	33.69
HM	3	**(a)**	24.49	8.77	17.88	34.43	21.15
MS	3	**(a)**	28.19	12.22	19.42	42.15	23.01
SH	3	**(a)**	25.08	1.55	23.62	26.71	24.92
SIS	3	**(a)**	36.88	8.26	27.39	42.46	40.79
N.H	3	**(a)**	3999.67	1924.68	2130	5975	3894.00
**Carbohydrates (µg/g FW)**							
AAP	3	**(a,b)**	17.09	13.75	2.84	30.28	18.15
HM	3	**(a)**	18.12	10.17	11.22	29.8	13.33
MS	3	**(a,b)**	67.66	14.01	53	80.91	69.07
SH	3	**(a,b)**	57.01	39.22	12.18	84.96	73.89
SIS	3	**(a,b)**	49.24	7.88	40.29	55.11	52.32
N.H	3	**(b)**	2406.67	511.28	1935	2950	2335

Significant differences between the groups are indicated by distinct letters and were found through a Kruskal–Wallis analysis combined with a Dun post-test (*p* < 0.05)

#### Carbohydrate contents

The highest median of carbohydrate content recorded was that of the natural environment, with a value of 2335 µg/g (Table 7).

In the synthetic media studied, the samples with the highest carbohydrate content were those from the SH medium, followed by those from the MS, SIS, and AAP media, with medians of 73.89, 69.07, 52.32, and 18.15 µg/g fresh weight, respectively, and finally the HM medium with a median of 13.33 µg/g fresh weight.

The Kruskal–Wallis test shows a significant effect of growth medium on the carbohydrate content of the studied species (H(5, *N* = 18) = 12.649, *p* = 0.027) and the Dunn’s post hoc test revealed that the samples from the natural environment have a statistically significant richness in these organic compounds compared to the samples from the HM medium, and there are no significant differences between the samples from AAP, MS, SH, and SIS.

#### Chlorophyll (a)

The highest average content of chlorophyll (a) was observed in the samples of the SH medium, with a value of 95.07 ± 22.14 µg/g (Table 8). The lowest levels were those of the samples of the AAP and natural habitat (7.22 ± 6.09 µg/g and 9.93 ± 2.08 µg/g, respectively). The average content of samples in the MS, HM and SIS media was 67.87 ± 33.45, 64.73 ± 10.06, 23.99 ± 9.33 µg/g, respectively.

**Table 8:** Variation of *L. minuta*’s chlorophyll (a), chlorophyll (b), total chlorophyll, and carotenoid content in different studied culture media

	***N***		***Mean***	***Std. dev***	***Min***	***Max***
***Chlorophyll (a) (µg/g FW)***						
AAP	3	***(a)***	7.22	6.09	1.22	13.39
HM	3	***(b,c)***	64.73	10.06	58.85	76.35
MS	3	***(b,c)***	67.87	33.45	30.06	93.62
SH	3	***(c)***	95.07	22.14	73.48	117.72
SIS	3	***(a,b)***	23.99	9.33	18.47	34.76
N.H	3	***(a)***	9.93	2.08	8.15	12.22
***Chlorophyll (b) (µg/g FW)***						
AAP	3	***(a)***	12.67	14.74	3.43	29.67
HM	3	***(a)***	22.16	12.38	11.13	35.55
MS	3	***(a,b)***	40.72	16.68	27.94	59.58
SH	3	***(a,b)***	41.01	17.05	22.22	55.48
SIS	2	***(a)***	13.42	15.97	2.13	24.71
N.H	3	***(b)***	74.11	14.73	60.4	89.68
***Total Chlorophyll (µg/g FW)***						
AAP	3	***(a)***	19.89	10.30	10.47	30.88
HM	3	***(a,b)***	86.87	14.55	70.1	96.12
MS	3	***(b)***	108.55	16.78	89.61	121.53
SH	3	***(b,c)***	136.04	38.84	95.68	173.16
SIS	3	***(a)***	32.67	23.25	17.69	59.45
N.H	3	***(c)***	198.19	38.29	174.13	242.35
***Carotenoids (µg/g FW)***						
AAP	3	***(a,b)***	50.80	20.31	27.35	62.6
HM	3	***(a,b)***	20.05	11.98	8.2	32.16
MS	3	***(b)***	34.68	7.31	28.68	42.83
SH	3	***(b)***	38.03	7.64	32.25	46.69
SIS	3	***(a)***	3.70	3.31	0.16	6.71
N.H	3	***(b)***	54.71	4.80	49.47	58.88

Significant differences between the groups are indicated by distinct letters and were found through a ANOVA (or Welch ANOVA) combined with a Tukey HSD post-hoc (or post-hoc Dunnett test) (*p* < 0.05)

The ANOVA test showed a significant effect of growth medium on chlorophyll (a) content (*F*(5, 12) = 12.760, *p* < 0.001), and the comparison using the Tukey HSD post-hoc test revealed that the samples of the SH medium had significantly higher levels of this pigment than the samples from the AAP and natural environment, and that there are no significant differences between the samples of the natural habitat, SIS, and AAP.

#### Chlorophyll (b)

The highest average content of chlorophyll b was observed in *L. minuta* tissues of the natural environment (Table 8). In vitro, the highest value was found in *L. minuta* tissues of the SH medium, with a value of 41.01 ± 17.05 µg/g. The average contents in the MS, HM, SIS and AAP media were 40.72 ± 16.68, 22.16 ± 12.38, 13.42 ± 15.97, and 12.67 ± 14.74 µg/g, respectively.

The ANOVA test shows a significant effect of the growth medium on the chlorophyll b content (*F*(5,11) = 6.614, *p* = 0.004) and the post-hoc HSD Tukey comparison revealed that the samples of the natural habitat had a significantly higher concentration of these green pigments than those of the AAP, HM and SIS media.

#### Total chlorophyll

The highest average chlorophyll content was observed in the samples of the natural environment, with a value of 198.19 ± 38.29 µg/g (Table 8). In vitro, the highest value was found in the samples grown in the SH medium, with a value of 136.04 ± 38.84 µg/g, followed by those grown in the MS (108.55 ± 16.78 µg/g), HM (86.87 ± 14.55 µg/g), SIS (32.67 ± 23.25 µg/g), and AAP (19.89 ± 10.30 µg/g) media. The ANOVA test showed a significant effect of culture medium on the chlorophyll content (*F*(5,12) = 19.324, *p* < 0.05) and the post-hoc HSD Tukey test revealed that the wild-type samples had a higher concentration than the synthetic medium samples, except for the samples grown in the SH medium, where there was no significant difference.

#### Carotenoids content

The highest average content of carotenoids was recorded in the samples of the natural environment with a value of 54.71 ± 4.80 µg/g fresh weight (Table 8).

In the synthetic media, the highest value was observed in the samples of the AAP medium, followed by those of SH, MS, and HM with contents of 50.80 ± 20.31, 38.03 ± 7.64, 34.68 ± 7.31, and 20.05 ± 11.98 µg/g respectively. The lowest content was that of the samples of SIS with a value of 3.70 ± 3.31 µg/g.

The Welch ANOVA test shows a significant effect of the culture medium on the species’ carotenoid content (*F*(5,5.379) = 34.527, *p* < 0.05). The comparison by the post-hoc Dunnett test shows that these contents in the samples of the SH and MS media are significantly higher than those of the samples of the SIS medium (*p* < 0.05) and are not significantly different from those of the samples of the natural environment and the AAP and HM media.

#### Ratio

##### The ratio of chlorophyll (a/b)

The ratio of chlorophyll (a/b) was found to be the lowest in samples from the natural environment (0.14 ± 0.02) (Table 9). In vitro, the lowest ratio of chlorophyll (a/b) was observed in the AAP media (1.61 ± 1.40), followed by MS (2.05 ± 1.44), SH (2.50 ± 0.70), HM (3.60 ± 1.84) and finally, the SIS which yielded the highest value (5.10 ± 5.22). A Welch ANOVA test revealed a significant effect of the culture medium on the ratio of chlorophyll (a/b) (F(5,3.891) = 4.613,*p* < 0.05). However, post-hoc comparisons using the Dunnett test did not show any significant differences between the groups.

**Table 9:** Variation of chlorophyll (a/b) ratio and carotenoids/total chlorophyll

	***N***		***Mean***	***Std. dev***	***Min***	***Max***
***Chlorophyll (a/b)***						
AAP	3	***(a)***	1.61	1.40	0.04	2.72
HM	3	***(a)***	3.60	1.84	1.66	5.30
MS	3	***(a)***	2.05	1.44	0.50	3.35
SH	3	***(a)***	2.50	0.70	2.07	3.31
SIS	2	***(a)***	5.10	5.22	1.41	8.79
N.H	3	***(a)***	0.14	0.02	0.11	0.16
***Carotenoids / Total Chlorophyll***						
AAP	3	***(a)***	3.16	2.45	1.49	5.98
HM	3	***(a)***	0.23	0.13	0.09	0.33
MS	3	***(a)***	0.33	0.13	0.25	0.48
SH	3	***(a)***	0.29	0.04	0.25	0.34
SIS	3	***(a)***	0.19	0.19	0.00	0.38
N.H	3	***(a)***	0.28	0.04	0.24	0.31

Significant differences between the groups based on ANOVA (in case of carotenoids /total chlorophyll) or Kruskal-Wallis analysis (chlorophyll (a/b)) (*p* < 0.05)

##### The ratio of (carotenoids /total chlorophyll)

The ratio of carotenoids to total chlorophyll was found to be the highest in samples from the AAP medium (3.16 ± 2.45) (Table 9), followed by MS (0.33 ± 0.13), SH (0.29 ± 0.04), HM (0.23 ± 0.13) and SIS (0.19 ± 0.19). The ratio of samples from the natural environment was found to be 0.28 ± 0.04. A Kruskal–Wallis test did not reveal any significant effect of culture medium on this ratio (H(5, *N* = 18) = 7.760, *p* = 0.170).

## Discussion

### Physico-chemical properties of the natural habitat of *L. minuta*

In this study, we investigated the physico-chemical properties of the natural habitat of *L. minuta*. Our results showed that the pH values (7.2–7.76) of the habitat were within the previously reported range for *L. minuta* growth [22, 23]. The conductivity values (998.33–1139 µS/cm) were also found to be within the optimal range for duckweed growth, as reported by previous studies [22, 24]. The measured orthophosphate concentrations (0.36–1.26 g/L) were higher compared to those observed in other water bodies where *L. minor* and *L. minuta* naturally grow [25, 26].

The salinity values (0.48–0.51 psu) confirmed that duckweeds only grow in freshwater environments [27]. The ammonium concentrations (0.52–3.73 g/L) were found to be high, indicating that duckweeds have a preference for high NH_4_^+^ concentrations [28].

The chemical oxygen demand (COD) values recorded at the studied stations were low, with a range of 10.45–13.22 mg/L. However, previous research [29] has shown that duckweeds are resilient in the presence of high organic matter concentrations, with a range of 500–750 mg/L COD.

### Morphophysiological analyses

The highest median value of RGRα(N) was recorded in fronds of SH and MS during the first phase with a value of 0.126 d^−1^, corresponding to a doubling time of 5 days. The lowest median values of RGRα(N) were observed in fronds of HM, with a value of 0.033 d^−1^ in the first phase, corresponding to doubling times of 21 days. These values are significantly lower than those reported in the literature for *L. minuta* and other duckweeds (0.1 ~ 0.5) [30, 31]. This may be explained by the relatively long duration of the experiment, as an increase in culture duration may lead to lower doubling time [32, 33].

The root length of duckweeds in SH was found to be significantly higher during both phases of the study, compared to MS. This difference may be attributed to the higher availability of nutrients in the SH medium, as indicated in [34]. Additionally, the presence of low-concentration auxins in SH, as reported in the same study, may also play a role in this observed difference.

The changes observed in the proliferation and the morphology of duckweed between the first and the second phase could be explained by the impact of the microbial communities of duckweed which is hardly observed in short term experiments under non-axenic conditions (frequency of detected infections by algae, bacteria and fungus in the first phase was statistically higher (data not shown)). In fact, some bacteria called plant growth-promoting bacteria (PGPB) have the abilities to increase the chlorophyll content and to increase the number of duckweed fronds*: Acidobacteria strains, Acinetobacter calcoaceticus, Aquitalea magnusonii H3, Bacillus amyloliquefaciens FZB42, Ensifer sp. SP4, Exiguobacterium sp. MH3, Pelomonas strains, Pseudomonas strains* …) [35–42]. However, others may negatively impact the growth and overall health of the duckweeds, leading to changes in their biomolecular composition (plant growth-inhibiting bacteria (PGIB) (*Acinetobacter ursingii M3, Asticcacaulis exentricus M6, Blastomonas natatorial M5* …) [40]. Furthermore, even some of the bacterial strain known to be PGPB could promote growth penalties in case of nutrient deficiency [43]. Moreover, the competition with duckweeds and algae (*Chlorella sp., Chlamydomonas sp*., …), in the first phase, is a severe threat for long-term cultivation [44–46]. These algae could reduce N, P, Fe and Mn concentrations of the medium drastically and increase the pH beyond 10 [45].

The established regression models indicate that in conditions of controlled aeration, the best medium to use is that of SH, followed by MS, which ensures an increase of more than 12% growth in each day of culture. This rate is much less significant (does not reach 5%) in conditions of uncontrolled aeration and in a culture of 15 days.

### Biochemical analyses

The results of this study suggest that the growth medium composition and the conditions under which the culture is performed can have a significant impact on the levels of proteins, carbohydrates, chlorophylls, and carotenoids present in the plant. Differences in starch and biomass production of duckweeds could be due to the differences in nitrogen sources in the used media (Ca(NO_3_)_2_ and KNO_3_ in HM, NH_4_NO_3_, and KNO_3_ in MS, …) as has been shown by [47]. High levels of chlorophyll b, total chlorophyll and carotenoids in the samples in natural environments, SH and MS media can be attributed to the effect of auxin and vitamin B on chlorophyll biosynthesis as has been reported by [48–50]. These findings are consistent with previous research in this area, indicating that the growth medium can play a crucial role in shaping the biomolecular content of duckweeds [3, 4, 27].

Taking into consideration the biochemical parameters of samples in natural habitat, our results highlight that the synthetic media tested in the present study can negatively impact the protein and total chlorophyll content of samples and further research based on artificial intelligence models (such as artificial neural networks (ANNs), decision trees, random forest (RF), and genetic algorithms (GA)) and optimization algorithms is needed to determine the specific conditions and synthetic media composition that best promote the growth and maintenance of this duckweed in long-term culture, while minimizing changes in its biomolecular composition.

## Conclusion

In this study, we have investigated the impact of different synthetic media on the growth, morphology, and biochemical composition of the duckweed species *L. minuta* in long-term cultivation. Our findings highlight the significance of growth medium composition and culture conditions on the levels of proteins, carbohydrates, chlorophylls, and carotenoids in duckweed. Among the tested media, Schenk-Hildebrand (SH) and Murashige-Skoog (MS) media were identified as the most suitable for in vitro culture of *L. minuta*.

However, our study has some limitations, as the proliferation rate of *L. minuta* is influenced by both the culture media composition and the presence or absence of infections. This may limit the generalizability of our findings to different environments or contexts. Addressing these limitations, we suggest future research to focus on utilizing artificial intelligence models and optimization algorithms for determining the specific conditions and synthetic media composition that promote optimal growth and maintenance of *L. minuta* in long-term culture while minimizing changes in its biomolecular composition.

The implications of our study emphasize the importance of understanding the effects of media composition on duckweed growth and biomolecule content. Gaining insights into these aspects is essential for unlocking the full potential of duckweed in various industries, such as animal feed, bioenergy production, and bioremediation. Moreover, understanding the complex relationships between duckweed and microbe associations is crucial for promoting sustainable aquatic ecosystem management and conservation.

## Data Availability

The datasets used and/or analysed during the current study available from the corresponding author on reasonable request.
